# Non-Classical Transformation of Benzendiazonium Hydrogen Sulfates. Access to 1,3-Dimethylisochromeno[4,3-*c*]pyrazol-5(1*H*)-one, a Potential Benzodiazepine Receptor Ligand

**DOI:** 10.3390/molecules181013096

**Published:** 2013-10-22

**Authors:** Benedetta Maggio, Demetrio Raffa, Maria Valeria Raimondi, Giuseppe Daidone

**Affiliations:** Dipartimento di Scienze e Tecnologie Biologiche Chimiche e Farmaceutiche, Università degli Studi di Palermo, Via Archirafi, 32, 90123-Palermo, Italy; E-Mails: benedetta.maggio@unipa.it (B.M.); mariavaleria.raimondi@unipa.it (M.V.R.); giuseppe.daidone@unipa.it (G.D.)

**Keywords:** heterocycles, Pschorr reaction, Sandmeyer reaction, 1,5-hydrogen atom transfer, isochromeno[4,3-*c*]pyrazol-5(1*H*)-one

## Abstract

The compound 2-((1,3-dimethyl-1*H*-pyrazol-5-yl)(methyl)carbamoyl)benzene-diazonium hydrogen sulfate (**10**) was reacted with copper sulfate and sodium chloride, in the presence of ascorbic acid as reducing agent, to afford a mixture of the chlorinated epimers 4′-chloro-2,2′,5′-trimethyl-2′,4′-dihydrospiro[isoindoline-1,3′-pyrazol]-3-one (**18**) and (**19**), the epimers 4′-hydroxy-2,2′,5′-trimethyl-2′,4′-dihydrospiro[isoindoline-1,3′-pyrazol]-3-one (**20**) and (**21**), and *N*-(1,3-dimethyl-1*H*-pyrazol-5-yl)benzamide (**22**). Under the foregoing conditions, diazonium salt **10** affords neither the 2-chloro-*N*-(1,3-dimethyl-1*H*-pyrazol-5-yl)-*N*-methylbenzamide (**23**) nor the tricyclic derivative **24**, the classical products of the Sandmeyer and Pschorr reactions, respectively. Finally, by heating **20** at 210 °C the compound 1,3-dimethylisochromeno[4,3-*c*]pyrazol-5(1*H*)-one (**24**) was obtained. The transformation under the above conditions of 2-((4-chloro-3-methyl-1-phenyl-1*H*-pyrazol-5-yl)(methyl)carbamoyl)benzendiazonium hydrogen sulphate (**11**) afforded 4′,4′-dichloro-2,5′-dimethyl-2′-phenyl-2′,4′-dihydrospiro[isoindoline-1,3′-pyrazol]-3-one (**29**) as the sole reaction product.

## 1. Introduction

**Scheme 1:**
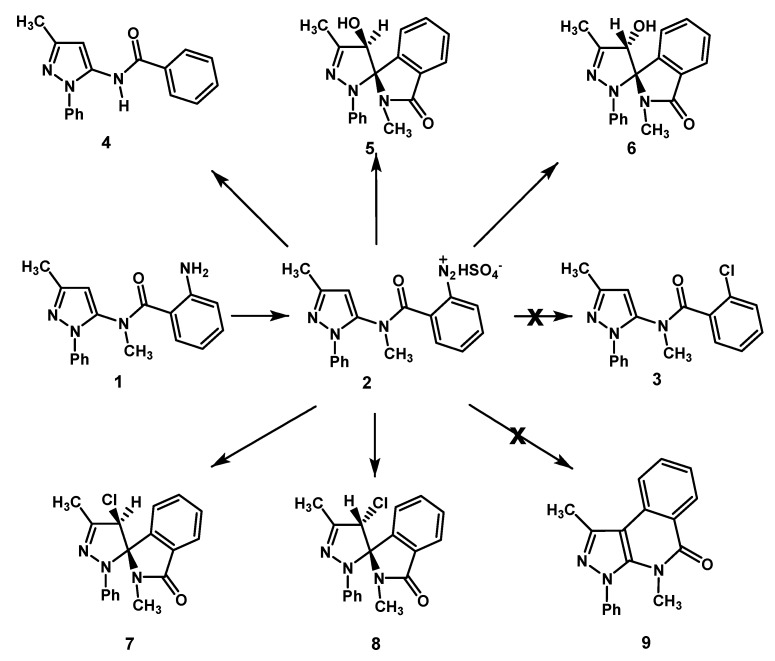
Transformation of the diazonium salt **2** by reaction with CuSO_4_/NaCl/ascorbic acid.

Previously we reported the transformation of diazonium hydrogen sulphate **2** derived from 2-amino-*N*-methyl-*N*-(3-methyl-1-phenyl-1*H*-pyrazol-5-yl)benzamide (**1**). This reaction was carried out with CuSO_4_ and NaCl, in the presence of ascorbic acid as a reducing agent [1] (Scheme 1). The above mixture was used earlier by Hanson and co-workers [2] to perform the Sandmeyer reaction on 4-chlorobenzendiazonium chloride in a homogeneous aqueous phase. Ascorbic acid reduces Cu(II) to Cu(I) which, in turn, reduces the diazonium ion to a diazenyl radical. The latter intermediate decomposes to dinitrogen and the phenyl radical. Copper(II) ions form complexes with chloride ions which are able to transfer a chloro radical to the 4-chlorophenyl by a ligand transfer process to afford 1,4-dichlorobenzene [2]. The diazonium hydrogen sulfate **2** afforded neither the chloro derivative **3**, the product of the classical Sandmeyer reaction, nor the tricyclic derivative **9**, the expected product of the competitive Pschorr ring closure, and instead we obtained epimers **7** and **8** [1].

**Figure 1:**
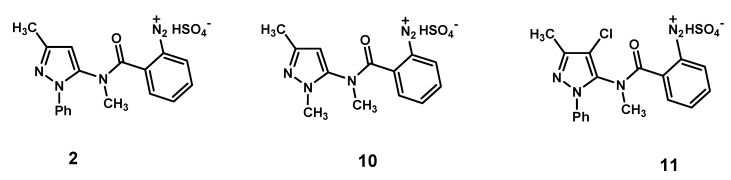
Diazonium hydrogen sulfates **2**, **10** and **11**.

Recently a re-investigation of the transformation reaction of diazonium salt **2** in the presence of CuSO_4_/NaCl/ascorbic acid has allowed to establish that a demethylation process also takes place to give *N*-(3-methyl-1-phenyl-1*H*-pyrazol-5-yl)benzamide **4** (for the mechanism see Scheme 5, below). Moreover, trace amounts of the hydroxy spiro derivatives **5** and **6** [1] were detected by TLC analysis. In our continuing research on this reaction [1,3,4,5], we became interested in investigating the behaviour of substrates possessing an alkyl group, such as methyl, in lieu of the pyrazole N-phenyl, as well as a substituent at the 4-position of the pyrazole nucleus, in order to verify whether such modifications are able to influence the course of the reaction. In fact, phenyl and methyl groups exert different electronic as well as steric effects on the pyrazole ring. Moreover, electronic and steric effects engendered by the substituent at the 4-position of the pyrazole nucleus could also affect the reaction outcome. Here, we describe the CuSO_4_/ascorbic acid-catalyzed decomposition in the presence of NaCl of the diazonium hydrogen sulfates **10** and **11** (Figure 1). These intermediates bear a methyl group or a chloro atom on the pyrazole nucleus, at positions 1 or 4, respectively.

## 2. Results and Discussion

**Scheme 2:**
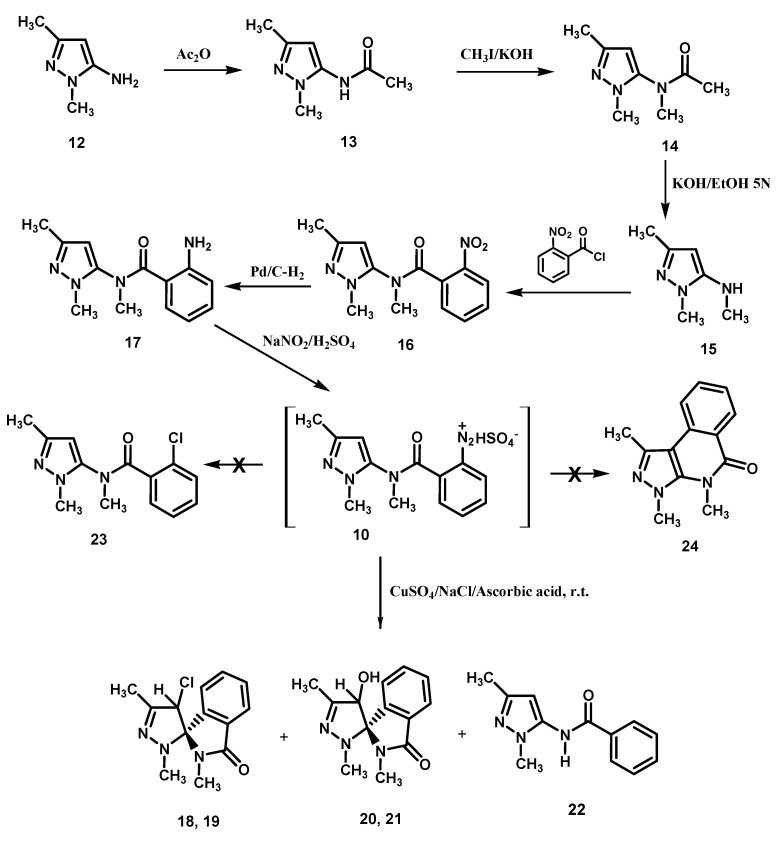
Preparation of the amino derivative **17** and its transformation.

**Scheme 3:**
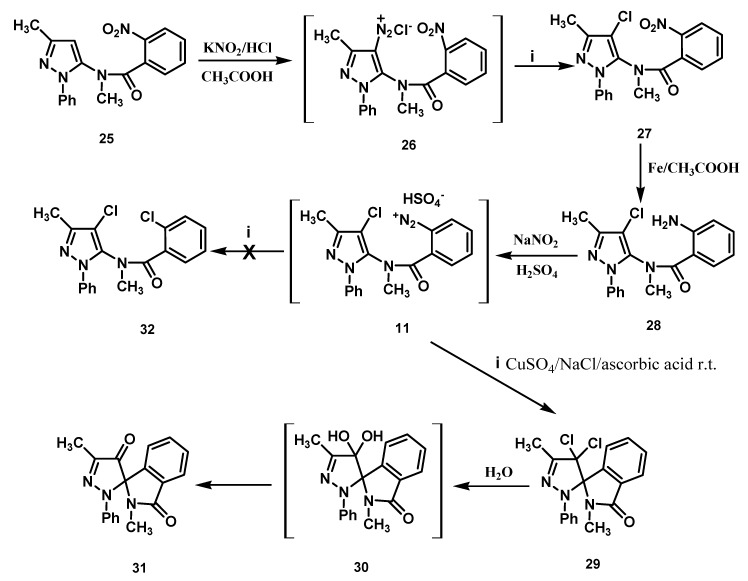
Preparation of the amino derivative **28** and its transformation.

Benzendiazonium hydrogen sulfates derivatives **10** and **11** were prepared following Scheme 2 and Scheme 3.

The amine **12** was reacted with acetic anhydride to give the acetamide derivative **13** which, in turn, was methylated with methyl iodide (Scheme 2). The obtained di-substituted acetamide derivative **14** was hydrolyzed with a potassium hydroxide ethanol-water solution to give the pyrazol-5-amine **15**.

Condensation of **15** with 2-nitrobenzoyl chloride afforded the 2-nitrobenzamide derivative **16** which was reduced with hydrogen in the presence of palladium/activated charcoal catalyst to give the 2-amino-*N*-methylbenzamide derivative **17**, diazotization of which produced the diazonium hydrogen sulfate **10**. Exposure of **10** to CuSO_4_/NaCl/ascorbic acid, as seen earlier for **2**, afforded a mixture of the chlorinated epimers (1*S*,4′*R*)- (or (1*S*,4′*S*)-) and (1*S*,4′*S*)- (or (1*S*,4′*R*)-) **18** and **19**, respectively, and the hydroxyl epimers (1*S*,4′*R*)- (or (1*S*,4′*S*)-) and (1*S*,4′*S*)- (or (1*S*,4′*R*)-) **20** and **21**, together obviously with the related enantiomers (not represented in the Schemes), and the benzamide derivative **22**. Finally, no evidence was obtained for the formation of the chloro derivative **23**, the product of the classical Sandmeyer reaction, as well as of 1,3,4-trimethylpyrazole[3,4-c]isoquinoline-5(4H)-one (**24**), the expected product of a possible competing Pschorr ring closure.

The preparation of substrate **11** started with the reaction of the 2-nitrobenzamide derivative **25** with a seven-fold excess of nitrous acid in acetic acid, resulting in the direct introduction of a diazo group at the 4-position of the pyrazole nucleus [6,7] (Scheme 3). The transformation *in situ* of the diazonium salt **26** into the chloro derivative **27** was performed with CuSO_4_, NaCl and ascorbic acid [1,2]. Compound **27** was then reduced with iron in acetic acid, and the aniline derivative **28** thus obtained was diazotized to furnish **11**.

The reaction of this diazonium salt under the same conditions employed earlier for **2** and **10** afforded the dichloro spiro derivative **29**, as the sole product of reaction (Scheme 3). Compound **29** was then converted into dione derivative **31**, possibly via intermediate **30**, upon refluxing in water. The new compounds were characterized by means of analytical and spectral data. The relative configuration of the pyrazoline C(4′)-atom of the spiro-compounds **18**–**21** were not determined. The work up of the reaction mixture obtained by transformation of **10** did not allow us to isolate the epimers **18** and **19**. The ^1^H-NMR spectrum of the obtained mixture showed singlets at 2.05, 2.07, 2.37, 2.44, 2.79 and 2.81 ppm, as well as at 5.54 and 5.83 ppm (ratio 1:2.25), consistent with the methyls and the pyrazoline H-4 of both epimers. On the contrary, epimers **20** and **21** were obtained as single compounds. The ^1^H-NMR spectrum of **20** showed three singlets at 1.94, 2.34 and 2.78 ppm, attributable to three methyls, and two doublets centered at 5.11 and 6.12 ppm for the pyrazoline H-4 and OH-4, respectively. Upon D_2_O exchange the OH signal in the the spectrum disappeared, and the H-4 doublet collapsed to a singlet. The IR spectrum confirmed the presence of the pyrazoline hydroxyl by the absorption band at 3,263 cm^−1^. The epimer **21** produced very similar IR and ^1^H-NMR spectra. The analogous compounds **22** and **4** were identified by comparison of their physical and spectroscopic data with authentic specimens (see the Experimental Section).

As regards **29**, the ^1^H-NMR spectrum showed signals at 2.38 and 2.66 ppm for two methyls, as well as those in the range 6.70–7.96 ppm for nine aromatic protons. The assigned structure of compound **29** was confirmed by its ^13^C-NMR spectrum and by its chemical modification. In fact, the action of water on compound **29** afforded a product which was identical in all respects (mixed melting point, TLC, MS, IR) to dione derivative **31** [1].

**Scheme 4:**
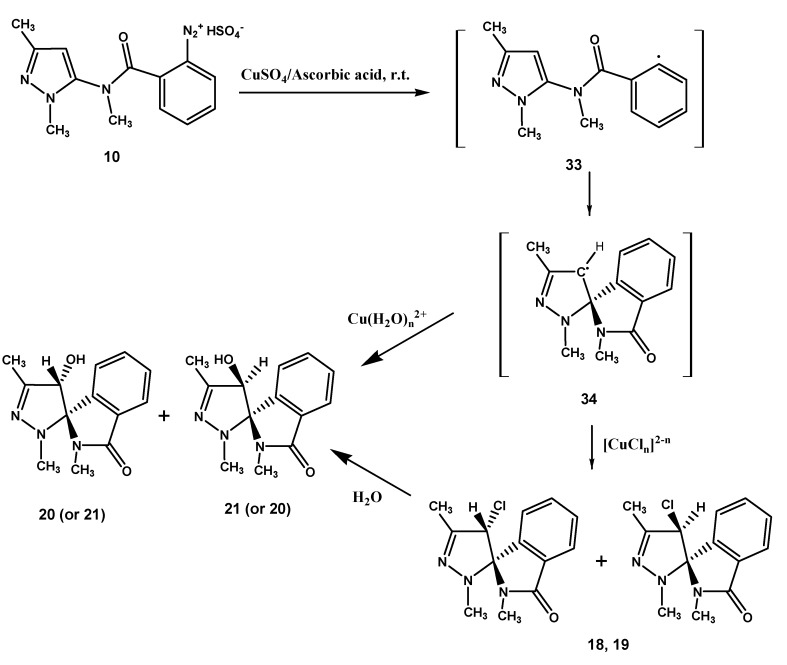
Suggested mechanism for the transformation of **10**.

**Scheme 5:**
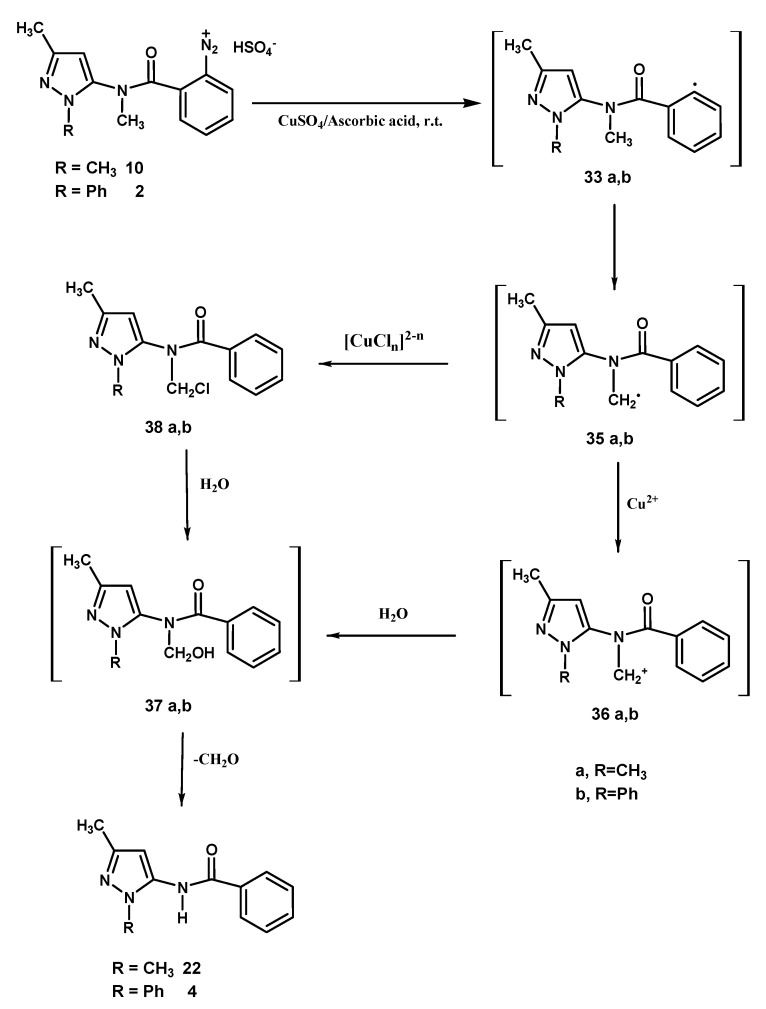
1,5-hydrogen atom transfer by -reaction in the transformation of diazonium salts **10**, **2**.

Rationalization of the formation of **18**, **19**, **20**, **21** and **4**, **22** is outlined in the Scheme 4 and Scheme 5. Formation of the chloro epimers **18** and **19** from **10** takes place *via* the intermediates **33** and **34** (Scheme 4). The latter transforms to **18** and **19** by a chloro transfer process from copper(II)-chloro complexes [1,2]. As regards the hydroxy spiro epimers **20** and **21**, they might be afforded by two different mechanisms: by transfer of H_2_O^+^ from the hydratation shell of the aqueous copper(II)-complex [8] to radical spiro intermediate **34**, or by nucleophilic replacement of the chloro atom in **18** and **19** by attack of a molecule of water. We noted that the chloro epimers **7**, **8** (Scheme 1) and **18**, **19** (Scheme 2) were obtained as precipitates from the solutions of diazonium salts **2** and **10** respectively, being the yield for **7**, **8** [1] quite higher than that of **18**, **19** (45% *versus* 8%). Moreover, extraction of the mother liquors of the above reaction mixtures of **2** and **10** allowed us to obtain hydroxy spiro epimers only in the case of **10** (that is **20** and **21**, yield 7%). Instead, only trace amounts of the hydroxy spiro epimers **5**, **6** could be detected by TLC in the crude mixture of **7**, **8**. We also observed that the mixture of **18** and **19** could be transformed in **20** and **21** when it was reacted with cold (5 °C) 0.09 M sulfuric acid solution, where **18**, **19** slowly dissolved, allowing the mixture to stand at r.t. for 1 h, mimicking thus the pH conditions of the reaction medium in which **10** transformed. When the same experiment was performed with the mixture of **7** and **8** no dissolution of the epimers in the acidic medium was observed. The suspension was extracted with ethyl acetate, and TLC of the extract did not reveal any transformation to give **5** and **6**. At this point we realized that the low yield for **18**, **19** is due to their solubility in the reaction medium, where they undergo a nucleophilic substitution by attack of a molecule of water to afford the hydroxy spiro epimers **20** and **21**. Nevertheless, a radical transfer process of H_2_O^+^ to the spiro intermediate **34** to give **20**, **21** can’t be ruled out (Scheme 4).

The latter process can be invoked for the formation of the trace amounts of **5** and **6**. Lastly, compounds **4** and **22** (Scheme 5) could be justified by considering a 1,5-hydrogen atom transfer process affording the radical intermediates **35a**,**b** which possibly undergo oxidation by cupric ions [9] to give carbocations **36a**,**b**. The oxidation process would probably be favoured by the resonance stability of **36a**,**b**, but the direct ligand radical transfer route from **35a**,**b** to **38a**,**b** cannot be ruled out. Intermediates **36a**,**b** and **38a**,**b** react with water affording the unstable compounds **37a**,**b** which by loss of formaldehyde transform into benzamide derivatives **22** and **4**, respectively.

Considering all the above data we concluded that the apparent different chemical behaviour between diazonium salts **2** and **10** is due to different solubilities of phenyl- and methyl-substituted chloro epimers, rather than to any electronic and steric effects of the *N*-phenyl- or *N*-methyl-pyrazole substituents.

**Scheme 6:**
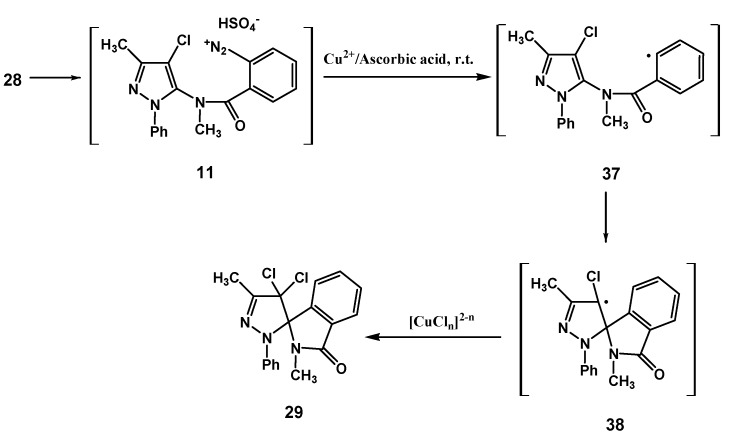
Suggested mechanism for the transformation of **11**.

As regards the transformation of the diazonium salt **11**, performed under the identical conditions followed for the chemical analogues **10** and **2**, the production of the dichloro spiro compound **29** was observed, which demonstrates that substitution at the 4-position of the pyrazoline nucleus does not hinder the ligand radical transfer process from the copper(II)-chloro complex to radical species **38** (Scheme 6).

**Scheme 7:**
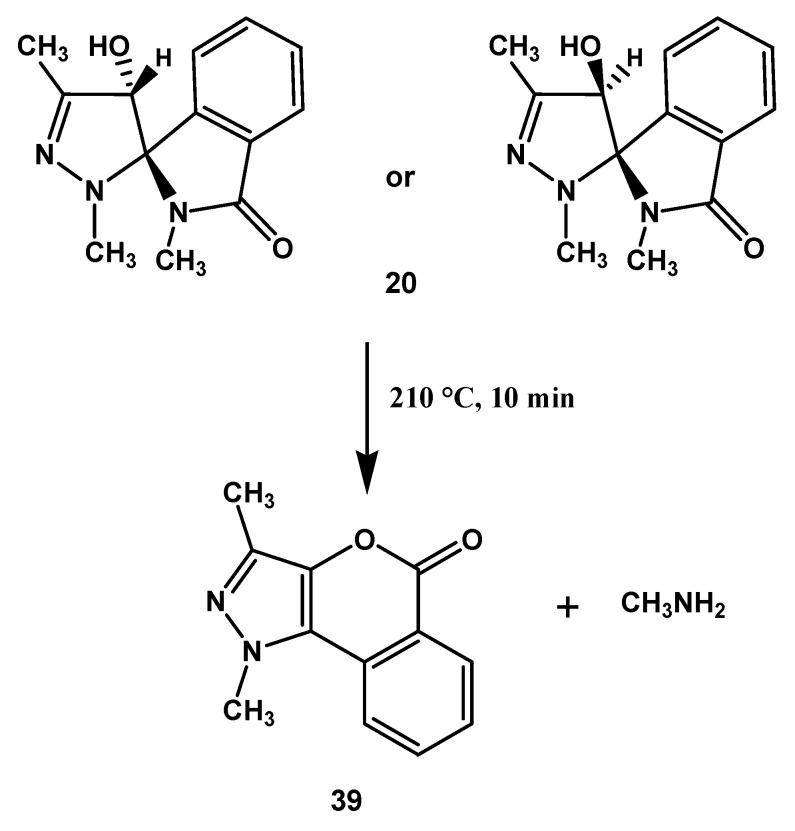
Transformation of **20** by fusion.

The spiro compound **20** proved to be a useful intermediate since it was transformed by heating at 210 °C into 1,3-dimethylisochromeno[4,3-*c*]pyrazol-5(1*H*)-one (**39**) (Scheme 7). The latter compound has the potential as a benzodiazepine receptor ligand. In fact, this compound shows two hydrogen bond acceptor atoms at the distance of about 3.5 Å, that is the isochromene oxygen and the nitrogen at position 2 of the pyrazole nucleus, which are mandatory in the molecule for a good affinity at the benzodiazepine binding site [10].

## 3. Experimental

### 3.1. General

Reaction progress was monitored by TLC on silica gel plates (Merck 60, F_254_, 0.2 mm). Organic solutions were dried over Na_2_SO_4_. Evaporation refers to the removal of solvent on a rotary evaporator under reduced pressure. All melting points were determined on a Büchi 530 capillary melting point apparatus and are uncorrected. IR spectra were recorded with a Perkin Elmer Spectrum RXI FT-IR System spectrophotometer as solid in KBr disc. ^1^H-NMR and ^13^C-NMR spectra were obtained in CDCl_3_ or DMSO-*d6* at 300.13 and 75.47 MHz respectively, using a Bruker AC series 300 MHz spectrometer (tetramethylsilane as an internal standard): chemical shifts are expressed in ppm values. Mass spectra at 70 eV were obtained using an Autospec Ultima Ortogonal T.O.F.T. (Micromass) spectrometer. Merck silica gel (Kiesegel 60/230-400 mesh) was used for flash chromatography columns. Microanalyses data (C, H, N) were obtained by an Elemental Vario EL. III apparatus and are within ±0.4% of the theoretical values. Yields refer to products after one crystallization. The names of the compounds were obtained using the Chem Draw 9.0.1 software of Cambridge Soft (Cambridge MA, USA).

### 3.2. Preparation of N-(1,3-Dimethyl-1H-pyrazol-5-yl)acetamide (**13**)

This compound was prepared following a literature method [11] modified by us: 1,3-dimethyl-1*H*-pyrazol-5-amine (**12**, 11.4 g, 0.102 mol) was reacted under stirring at r.t. with acetic anhydride (45 mL) for 24 h. After this time the solution was evaporated to give an oily residue which solidified when treated with triturated ice (20 g) and scraped. After filtration the material was air dried and crystallized from ethyl acetate/petroleum ether (b.p. 40–70 °C) to give **13** in 70% yield; mp: 43–45 °C; IR (KBr, cm^−1^): 3,123 (broad, NH), 1,667 (CO); ^1^H-NMR (DMSO-*d6*, ppm): 2.04 (3H, s, CH_3_); 2.07 (3H, s, CH_3_); 3.55 (3H, s, CH_3_); 5.95 (1H, s, pyrazole H-4); 9.84 (1H, s, exchangeable with D_2_O, NH); Anal. Calcd. for C_7_H_11_N_3_O (153.18) : C, 54.89%; H, 7.24%; N, 27.43%. Found: C, 54.51%; H, 6.86%; N, 27.28%.

### 3.3. Preparation of N-(1,3-Dimehyl-1H-pyrazol-5-yl)-N-methylacetamide (**14**)

To a solution of *N*-(1,3-dimethyl-1*H*-pyrazol-5-yl)acetamide (**13**, 1 g, 6.5 mmol) in hot acetone (26 mL) was added KOH (1.58 g), and the mixture was refluxed for 10 min. After this time CH_3_I (0.67 mL, 10 mmol) in acetone (4 mL) was added and reflux was continued for one hour. The mixture was filtered, and evaporation of the filtrate afforded a residue which was treated with water (20 mL) and extracted with diethyl ether (4 × 20 mL). The combined ether extracts were evaporated to give a solid residue which was crystallized from ethyl acetate affording compound **14** as colorless crystals in 46% yield, mp: 128–130 °C. MS (*m/z*): 167 (M^+^); IR (KBr, cm^−1^): 1,681 (CO); ^1^H-NMR (DMSO-*d6*, ppm): 1.75 (3H, s, CH_3_); 2.12 (3H, s, CH_3_); 3.03 (3H, s, CH_3_); 3.57 (3H, s, CH_3_); 6.04 (1H, s, pyrazole H-4). ^13^C-NMR (DMSO-*d6*) (ppm): 13.79 (CH_3_), 21.39 (CH_3_), 34.54 (CH_3_), 35.39 (CH_3_), 101.50 (CH), 141.83 (C), 146.30 (C), 169.82 (CO); Anal. Calcd. for C_8_H_13_N_3_O (167.21) : C, 57.46%; H, 7.84%; N, 25.13%. Found: C, 57.21%; H, 7.90%; N, 24.76%.

### 3.4. Preparation of N,1,3-Trimethyl-1H-pyrazol-5-amine (**15**) [12]

To a solution of N-(1,3-dimethyl-1*H*-pyrazol-5-yl)-*N*-methylacetamide (**14**, 7.16 g, 42 mmol) in ethanol (69 mL) was added a 5 N aqueous solution of KOH (40 mL), and the mixture was refluxed for 10 h. After this time the reaction mixture was first filtered and then evaporated under vacuum to give a residue which was extracted with diethyl ether (4 × 90 mL). The combined extracts were evaporated under reduced pressure and the obtained residue was processed by flash chromatography [13]: column external diameter 4 cm, silica gel 0.040–0.063 mm, ethyl acetate/acetone (1:1 v/v ) as eluent (1.5 L), fractions each 50 mL. The first ten fractions were discarded, and fractions 11-25 were evaporated affording pure **15** as a pale yellow oil in 70% yield**.** IR (KBr, cm^−1^): 3,228 (NH); ^1^H-NMR (CDCl_3_, ppm): 2.18 (3H, s, CH_3_), 2.80 (3H, s, CH_3_), 3.53 (4H, s, CH_3_, NH; 3H after exchange with D_2_O), 5.27 (1H, s, pyrazole H-4); Anal. Calcd. for C_6_H_11_N_3_ (125.17) : C, 57.57%; H, 8.86%; N, 33.57%. Found: C, 57.21%; H, 8.86%; N, 33.53%.

### 3.5. Preparation of N-(1,3-dimethyl-1H-pyrazol-5-yl)-N-methyl-2-nitrobenzamide (**16**)

A solution containing equimolar amounts (6.16 mmol) of *N*,1,3-trimethyl-1*H*-pyrazol-5-amine (**15**) and 2-nitrobenzoyl chloride in dry chloroform (29 mL) was refluxed for 5 h. After the first hour triethylamine (0.86 mL) was added in four portions (0.43 mL; 0.21 mL; 2 × 0.11 mL respectively, with intervals of 1 h between additions). The solution was evaporated under reduced pressure, the residue washed with cold water and extracted with dichloromethane (3 × 50 mL). The combined extracts were dried (sodium sulfate) and the solid residue afforded by evaporation was crystallized from ethyl acetate to give **16**, as colorless crystals, in 64% yield, mp 132–133 °C; MS (*m/z*) 274 (M^+^); IR (KBr, cm^−1^): 1,671 (CO); ^1^H-NMR (CDCl_3_, ppm): 2.03 (3H, s, CH_3_), 3.40 (3H, s, CH_3_), 3.63 (3H, s, CH_3_), 5.70 (1H, s, pyrazole H-4), 7.29–8.30 (4H, a set of signals, C_6_H_4_); ^13^C-NMR (DMSO-*d6*, ppm): 13.92 (CH_3_), 35.35 (CH_3_), 36.36 (CH_3_), 102.44 (CH), 124.80 (CH), 128.67 (CH), 131.09 (CH), 132.34 (C), 134.96 (CH), 140.62 (C), 145.49 (C), 146.50 (C), 167.31 (CO); Anal. Calcd. for C_13_H_14_N_4_O_3_ (274.28) : C, 56.93%; H, 5.14%; N, 20.43%. Found: C, 56.75%; H, 5.13%; N, 20.62%.

### 3.6. Preparation of 2-Amino-N-(1,3-Dimethyl-1H-pyrazol-5-yl)-N-methylbenzamide (**17**)

To a solution of *N*-(1,3-dimethyl-1*H*-pyrazol-5-yl)-*N*-methyl-2-nitrobenzamide (**16**, 3.84 g, 14 mmol) in ethanol (150 mL) was added 10% palladium on activated charcoal catalyst (380 mg). The mixture was reacted with hydrogen in a Parr apparatus at 50 psi for 20 h. After this time the reaction mixture was filtered, and the filtrate was evaporated under vacuum to give an oily residue which was processed by Flash chromatography [13]: column external diameter 4.5 cm, silica gel (0.040-0.063 mm), ethyl acetate as eluent (1.8 L), fractions each 50 mL. The first 17 fractions were discarded. Fractions 18–21 were collected and evaporated under vacuum to afford **17** as a pale yellow pure oil in 44% yield; MS (*m/z*): 244 (M^+^); IR (KBr) (cm^−1^): 3490–3200 (multiple bands, NH_2_), 1638 (CO); ^1^H-NMR (CDCl_3_, ppm): 2.16 (3H, s, CH_3_), 3.34 (3H, s, CH_3_), 3.44 (3H, s, CH_3_), 4.81 (2H, s, NH_2_, exchangeable with D_2_O), 5.87 (1H, s, pyrazole H-4), 6.40–7.07 (4H, a set of signals, C_6_H_4_); Anal. Calcd. for C_13_H_16_N_4_O (244.29) : C, 63.91%; H, 6.60%; N, 22.93%. Found: C, 64.18%; H, 6.63%; N, 22.67%.

### 3.7. Preparation of 2-((1,3-Dimethyl-1H-pyrazol-5-yl)(methyl)carbamoyl)benzendiazonium hydrogen sulfate (**10**)

The pulverized amine **17** (2.07 g, 8.48 mmol) was dissolved in cooled (0–5 °C) aqueous sulfuric acid (5 N) (17 mL), and aqueous sodium nitrite (2.5 M) (3.5 mL) was added dropwise to the stirred solution. The solution was stirred for a further 15 min in the ice bath and was then checked for excess nitrous acid with potassium iodide starch paper; the eventual excess can be destroyed by addition of urea. This solution was utilized in the next step.

### 3.8. Transformation of the Diazonium Hydrogen Sulphate (**10**)

To a cold (0–5 °C) solution. (400 mL) of CuSO_4_**·**5 H_2_O (0.3 M) and NaCl (0.75 M), first the soln. of **10**, obtained from the previous procedure, and then ascorbic acid (370 mg, 2.11 mmol) were added under stirring. The mixture was stirred for 1 h at r.t. and then filtered. The solid product obtained was washed with cold water, dried in desiccator (anhydrous CaCl_2_, 24 h) and then crystallized from diethyl ether to give the epimeric mixture of (1*S*,4′*R*)- (or (1*S*,4′*S*)-) and (1*S*,4′*S*)- (or (1*S*,4′*R*)-)4′-chloro-2,2′,5′-trimethy-2′,4′-dihydrospiro[isoindoline-1,3′-pyrazol]-3-one (**18**) and **19**, respectively in 8% overall yield; GLC-MS: two peaks, 228 (*m/z*) for each, (M^+^-Cl); IR (KBr, cm^−1^): 1,710 (CO); ^1^H-NMR (DMSO-*d6*, ppm): 2.05 (3H, s, CH_3_), 2.07 (3H, s, CH_3_), 2.37 (3H, s, CH_3_), 2.44 (3H, s, CH_3_), 2.79 (3H, s, CH_3_), 2.81 (3H, s, CH_3_), 5.54 (1H, s, pyrazoline H-4); 5.83 (1H, s, pyrazoline H-4); 7.59–7.77 (2 × 4H, a set of signals, 2 × C_6_H_4_); Anal. Calcd. for C_13_H_14_ClN_3_O (263.72) : C, 59.21%; H, 5.35%; N, 15.93%. Found: C, 59.28%; H, 5.33%; N, 15.93%.

The mother liquors were saturated with NaCl and extracted with diethyl ether (3 × 100 mL). The combined extracts were dried (Na_2_SO_4_) and evaporated under reduced pressure. The oily residue (850 mg) was chromatographed following the flash procedure [13]: external diameter of the column 4.5 cm, silica gel (0.040–0.063 mm), ethyl acetate as eluent (2L), fractions each 50 mL. The initial seven fractions were discarded. Fractions 8 and 9 were evaporated under vacuum to give 130 mg (7%) of pure (1*S*,4′*R*)- (or (1*S*,4′*S*)-)4′-hydroxy-2,2′,5′-trimethyl-2′,4′-dihydrospiro[isoindole-1,3′-pyrazol]-3(2*H*)-one (**20**) as a colourless solid, mp 160–163 °C (ethyl acetate); MS (*m/z*): 245 (M^+^), 214 (M^+^-CH_3_NH_2_); IR(KBr, cm^−1^): 3,263 (OH), 1,671 (CO); ^1^H-NMR (DMSO-*d6*, ppm): 1.94 (3H, s, CH_3_), 2.34 (3H, s, CH_3_), 2.78 (3H, s, CH_3_), 5.11 (1H, d, *J* = 6.3 Hz, pyrazoline H-4), 6.12 (1H, d, *J* = 6.3 Hz, OH, exchangeable with D_2_O), 7.52-7.70 (4H, a set of signals, C_6_H_4_); Anal. Calcd. for C_13_H_15_N_3_O_2_ (245.28): C, 63.66%; H, 6.16%; N, 17.13%. Found: C, 63.42%; H, 6.55%; N, 17.02%.

The combined fractions 11–14 when evaporated gave a residue (170 mg) which was chromatographed by preparative TLC on silica gel (20 × 20 cm, thickness 2 mm; ethyl acetate/chloroform 1:1 as eluent). The plate showed three bands (U.V. light at 254 nm) among which the intermediate one underwent extraction with ethyl acetate. The extract was evaporated under vacuum and the residue obtained was crystallized to give a compound which was identical in all respects (mp, mixed mp, Rf, IR, ^1^H-NMR) with an authentic specimen of *N*-(1,3-dimethyl-1*H*-pyrazol-5-yl)benzamide (**22**) [7], 2% yield. The mother liquors from the crystallization of **22** were evaporated under vacuum, and the residue was processed by preparative TLC: 20 × 20 cm, thickness 2 mm, ethyl acetate/chloroform 1:1 as eluent. The crude product obtained underwent the same procedure to give 10 mg of pure (1*S*,4′*S*)- (or (1*S*,4′*R*)-)4′-hydroxy-2,2′,5′-trimethy-2′,4′-dihydrospiro[isoindoline-1,3′-pyrazol]-3-one (**21**), as a colorless solid, mp 177–180 °C; MS (*m/z*): 245 (M^+^), 214 (M^+^-CH_3_NH_2_); ^1^H-NMR (DMSO-*d6*, ppm): 1.97 (3H, s, CH_3_), 2.22 (3H, s, CH_3_), 2.85 (3H, s, CH_3_), 4.92 (1H, d, *J* =5.7 Hz, pyrazoline H-4), 5.85 (1H, d, *J* = 6.3 Hz, pyrazoline OH-4, exchangeable with D_2_O), 7.43-7.70 (4H, a set of signals, C_6_H_4_); ^13^C-NMR (DMSO-*d6*, ppm): 12.75 (CH_3_), 24.74 (CH_3_), 34.02 (CH_3_), 77.76 (CH), 91.86 (C), 111.21 (C), 122.10 (CH), 124.65 (CH), 128.83 (CH), 130.45 (CH), 131.52 (C), 139.47 (C), 152.38 (C), 166.02 (CO); Anal. Calcd. for C_13_H_15_N_3_O_2_ (245.28) : C, 63.66%; H, 6.16%; N, 17.13%. Found: C, 63.45%; H, 6.17%; N, 17.20%.

### 3.9. Preparation of 1,3-Dimethylisochromeno[4,3-c]pyrazol-5(1H)-one (**39**)

Compound **20** (30 mg) was melted at 210 °C for 10 min. The obtained material was crystallized from ethanol to give **39**, as colorless solid, in 69% yield, mp 237–239 °C; MS (*m/z*): 214 (M^+^); IR (KBr, cm^−1^): 1715 (CO); ^1^H-NMR (CDCl_3_, ppm): 2.37 (3H, s, CH_3_), 4.23 (3H, s, CH_3_), 7.55–8.46 (4H, a set of signals, C_6_H_4_); ^13^C-NMR (CDCl_3_, ppm): 9.96 (CH_3_), 40.07 (CH_3_), 119.86 (C), 120.47 (CH), 122.41 (C), 128.10 (CH), 128.58 (C), 132.47 (CH), 132.91 (C), 134.97 (CH), 136.95 (C), 162.08 (CO); Anal. Calcd. for C_12_H_10_N_2_O_2_ (214.22) : C, 67.28%; H, 4.71%; N, 13.08%. Found: C, 67.56%; H, 4.87%; N, 13.09%.

### 3.10. Preparation of N-(3-Methyl-1-phenyl-1H-pyrazol-5-yl)benzamide (**4**)

From the transformation of the diazonium salt **2** by the CuSO_4_/NaCl/ascorbic acid reagent combination: the diazonium hydrogen sulfate **2** derived from compound **1** [1] (5.9 mmol) was decomposed following the procedure used for **10**. The suspension obtained was filtered, and the solid was crystallized from ethanol (95% V/V) to give epimers **7**, **8**. The mother liquors when evaporated leave a residue (350 mg) which was processed by preparative TLC (two plates, 20 × 20 cm, thickness 2 mm, ethyl acetate/petroleum ether 3:7 as eluent). Work up allowed us to obtain a product (140 mg) which was identical in all respects (mp, mixed mp, IR, ^1^H-NMR) to an authentic specimen of compound **4** [7].The aqueous mother liquors were saturated with sodium chloride and extracted with ethyl acetate (4 × 150 mL). The combined extracts were evaporated to give a residue (70 mg) which was processed by preparative TLC. More of compound **4** (20 mg) was obtained following the above procedure, overall yield 10%.

### 3.11. Preparation of N-(4-Chloro-3-methyl-1-phenyl-1H-pyrazol-5-yl)-N-methyl-2-nitrobenzamide (**27**)

This compound was prepared by modifying the procedure reported in reference [14]. Compound **25** [15] (2 g, 5.95 mmol) was dissolved in acetic acid (57 mL) and then concentrated hydrochloric acid (36.5% w/w, 4.3 mL) was added. To the stirred solution a potassium nitrite aqueous solution (3.5 g of KNO_2_ in 1.8 mL of H_2_O) was added dropwise and stirring was continued for a further 24 h. The obtained suspension was filtered and the filtrate was treated first with an aqueous solution (300 mL) of hydrochloric acid (0.24 M), copper sulphate pentahydrate (0.3 M) and sodium chloride (0.75 M) and then with ascorbic acid (260 mg, 1.48 mmol). The mixture was stirred for 1 h at r.t. and then filtered. The solid product obtained was crystallized from ethanol (95% V/V) to give **27** (yield 60%) identical in all respects (mixed melting point, TLC, MS, IR) to an authentic specimen of compound **27** [14].

### 3.12. Preparation of 2-Amino-N-(4-chloro-3-methyl-1-phenyl-1H-pyrazol-5-yl)-N-methylbenzamide (**28**)

A suspension of Fe filings (12.41 g) in 5% (V/V) aqueous AcOH (16 mL) was heated at about 100 °C under stirring on the water bath of a rotavapor until H_2_ evolution ceased. The nitro derivative **27** (6.9 g, 18.5 mmol) was added in four portions, with intervals of 20 min. between additions. After the last addition, stirring was continued at 100 °C for 1 h, then the mixture was cooled to r.t. The pH of the suspension was adjusted to 7–8 with a saturated NaHCO_3_ solution. The solid was separated by filtration, air dried overnight, and then extracted with boiling CHCl_3_ (3 × 30 mL). The combined extracts were evaporated under vacuum, leaving an residue which was crystallized from ethyl acetate to give **28** in 80% yield, mp 165–166 °C; MS (*m/z*): 342 (M^+^); IR (cm^−1^): 3485, 3358 (NH_2_), 1640 (CO); ^1^H-NMR (ppm): 2.26 (3H, s, Me), 3.40 (3H, s, Me); 4.33 (2H, s, exchangeable with D_2_O, NH_2_), 6.40–7.35 (9H, m, C_6_H_5_ and C_6_H_4_); Anal. Calcd. for C_18_H_17_ClN_4_O (340.81) : C, 63.44%; H, 5.03%; N, 16.44%. Found: C, 63.35%; H, 5.21%; N, 16.56%.

### 3.13. Preparation of 2-((4-Chloro-3-methyl-1-phenyl-1H-pyrazol-5-yl)(methyl)carbamoyl)benzendiazonium Hydrogen sulphate (**11**)

The pulverized amine **28** (1.5 g, 4.41 mmol) was dissolved in cooled (0–5 °C) aqueous sulfuric acid (10 M) (17.6 mL) and aqueous sodium nitrite (2.5 M) (1.82 mL) was added drop-wise to the stirred solution. The solution was stirred for a further 15 min in the ice bath and was then checked for excess nitrous acid with potassium iodide starch paper. The eventual excess can be destroyed by addition of urea.

### 3.14. Decomposition of the Diazonium Hydrogen Sulphate **11**

To a cold (0–5 °C) soln. (220 mL) of CuSO_4_**·**5H_2_O (0.3 M) and NaCl (0.75M), first the soln. of **11** obtained from the previous procedure, and then ascorbic acid (195 mg, 1.11 mmol) were added under stirring. The mixture was stirred for 1 h at r.t. and then filtered. The solid product obtained was dried in desiccator (anhydrous CaCl_2_) for 24 h and then crystallized from ethyl acetate to give compound **29** in 80% yield, mp 202–203 °C; MS (*m/z*): 359 (M^+^); IR (cm^−1^):1713 (CO); ^1^H-NMR (CDCl_3_, ppm): 2.38 (3H, s, Me), 2.66 (3H, s, Me), 6.70–7.96 (9H, m, C_6_H_5_ and C_6_H_4_); ^13^C-NMR (CDCl_3_, ppm): 11.13 (CH_3_), 26.64 (CH_3_), 96.61 (C), 93.23 (C), 115.59 (2xCH), 122.45 (CH), 124.30 (CH), 127.21 (CH), 129.05 (2xCH), 130.98 (CH), 131.62 (CH), 132,66 (C), 139.08 (C), 142.21 (C), 147 (C), 167 (CO); Anal. Calcd. for C_18_H_15_Cl_2_N_3_O (359.06) : C, 60.01%; H, 4.20%; N, 11.66%. Found: C, 60.20%; H, 4.58%; N, 11.69%.

### 3.15. Reaction of 4′,4′-Dichloro-2,5′-dimethyl-2′-phenyl-2′,4′-dihydrospiro[isoindoline-1,3′-pyrazol]-3-one (**29**) with Water

To a solution of **29** (10 mg) in acetonitrile (1 mL) was added water (1 mL) and the mixture was refluxed for 30 min. The solution was evaporated under vacuum to give a pure residue which was identical in all respects to an authentic specimen of compound **31** [1].

## 4. Conclusions

The diazonium salts **2** and **10** show as unique structural diversity the different substituent at the 1-position of the pyrazole nucleus, that is phenyl or methyl. The above diversity does not lead to a different chemical reactivity of these diazonium salts when they are reacted with the reagents copper sulphate/ascorbic acid/sodium chloride. In fact, the observed difference in the composition of the reaction mixtures of **10** and **2** is not due to different electronic and/or steric effects of the pyrazole substituents at 1-position, but rather to a remarkable differential solubility between the pairs of epimers **18**, **19** and **7**, **8**. Epimers **18**, **19** partially dissolve in the reaction mixture and undergo nucleophilic replacement of the chloro atom to give the hydroxy spiro derivatives **20**, **21**, whereas epimers **7**, **8** do not follow this pathway. As regards diazonium salt **11**, the presence of a substituent at the 4-position of the pyrazole moiety does not hinder this position, and a dichloro spiro derivative **29** is obtained.

## Supplementary Materials

^1^H-NMR and ^13^C-NMR of compounds **17**, **18**, **19** (**mixture**), **20**, **21**, **28**, **29** and **39**. These materials can be accessed at: http://www.mdpi.com/1420-3049/18/10/13096/s1.
