# Supplementary material for: Non-Classical Transformation of Benzendiazonium Hydrogen Sulfates. Access to 1,3-Dimethylisochromeno[4,3-c]pyrazol-5(1H)-one, a Potential Benzodiazepine Receptor Ligand
Source: Molecules. 2013 Oct 22;18(10):13096–110. doi: 10.3390/molecules181013096 (PMC6270027; doi:10.3390/molecules181013096)

Supplementary Materials

Figure S1. ^{1}H-NMR spectrum of compounds **17**.

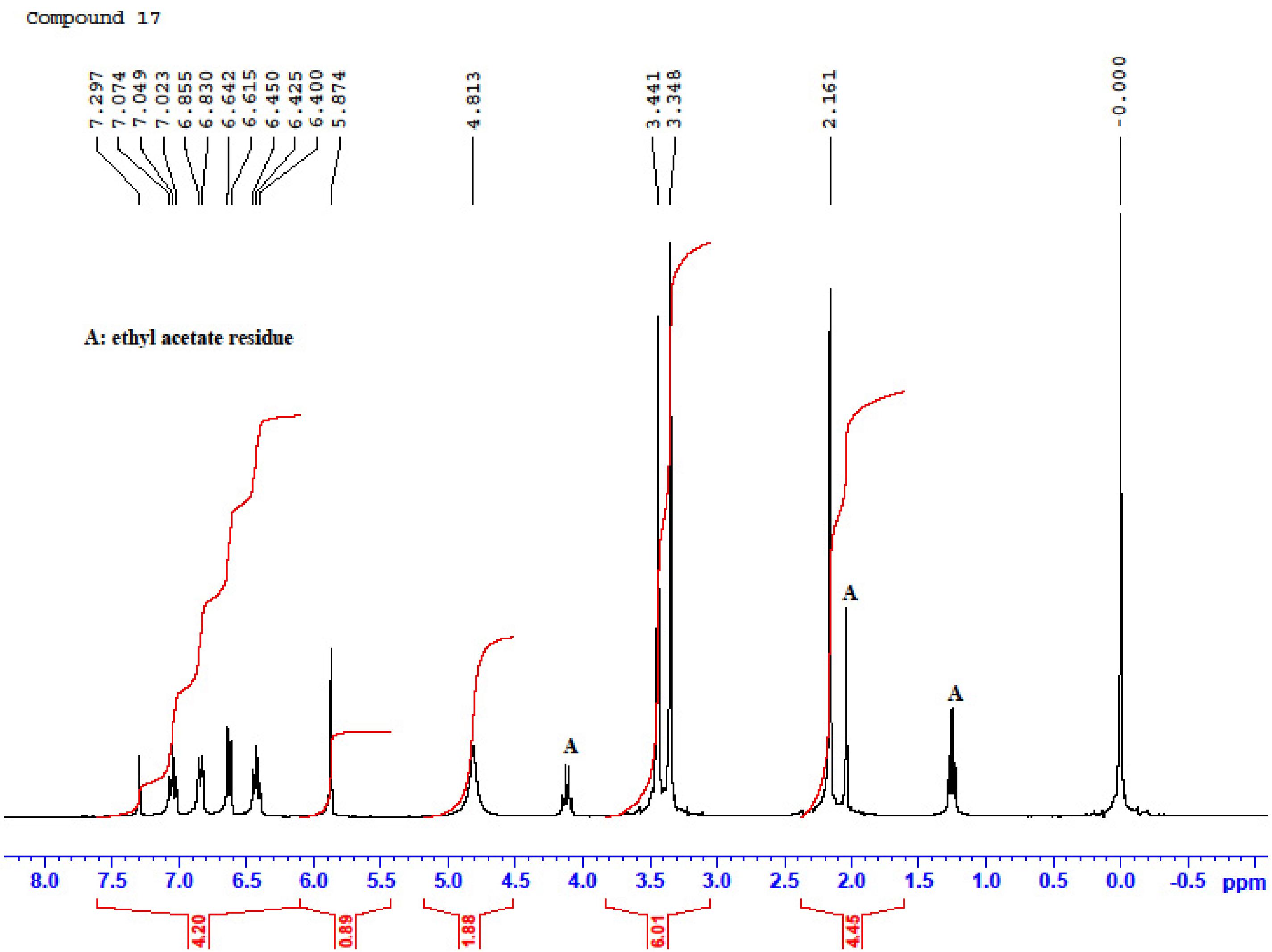

Figure S2. ^{1}H-NMR spectrum of compounds **18, 19**.

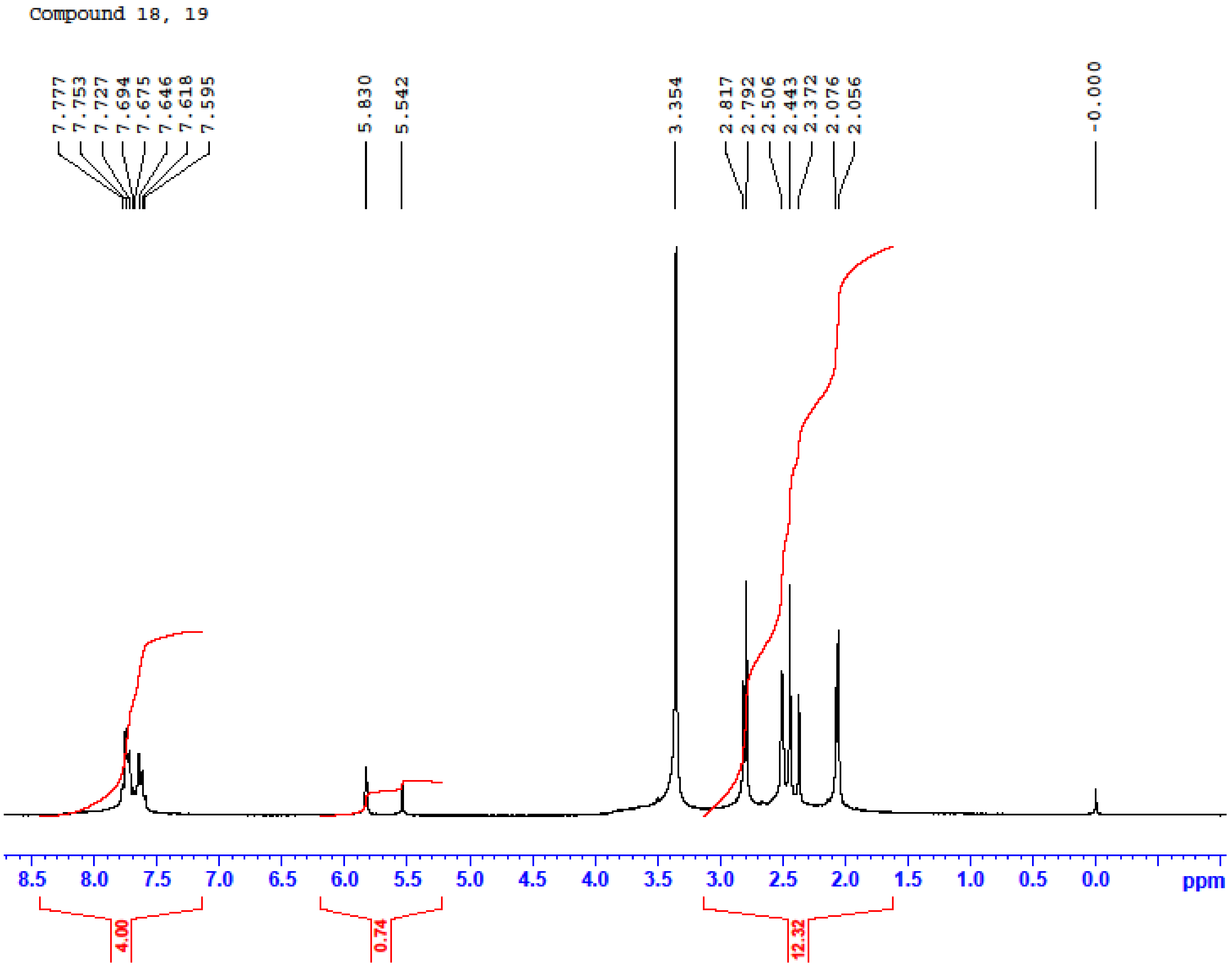

Figure S3. ^{1}H-NMR spectrum of compounds **20**.

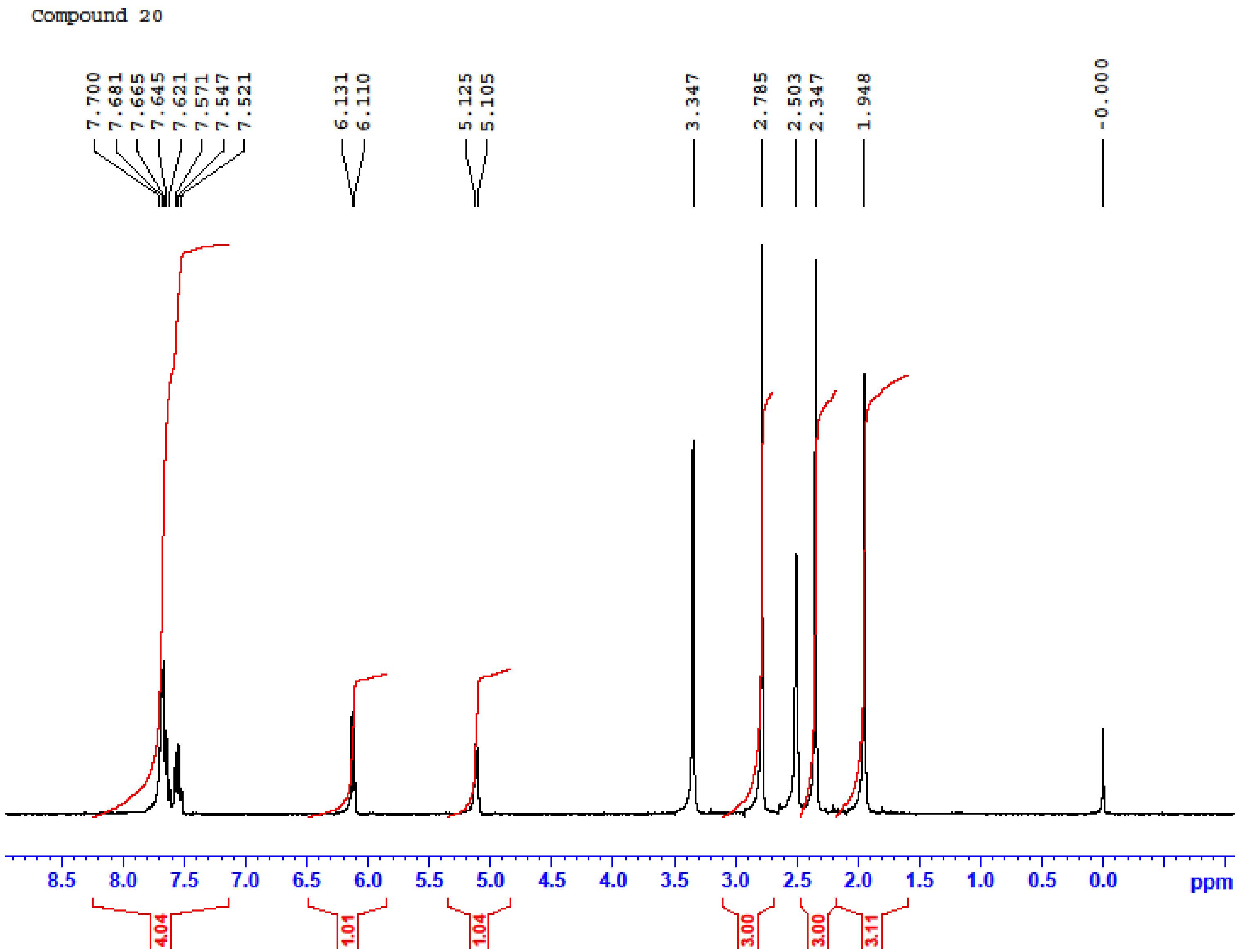

Figure S4. ^{1}H-NMR spectrum of compounds **21**.

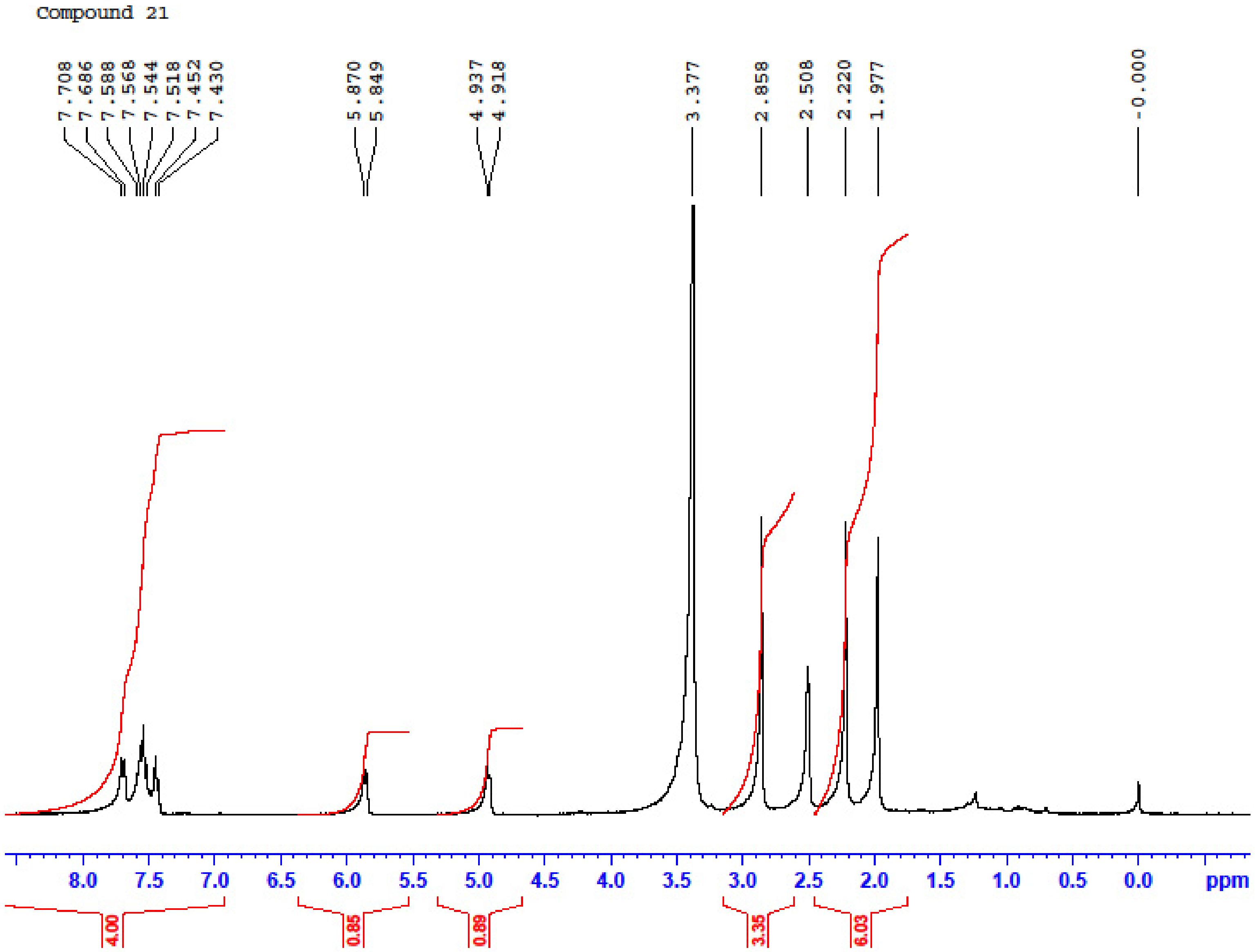

Figure S5. ^{1}H-NMR spectrum of compounds **28**.

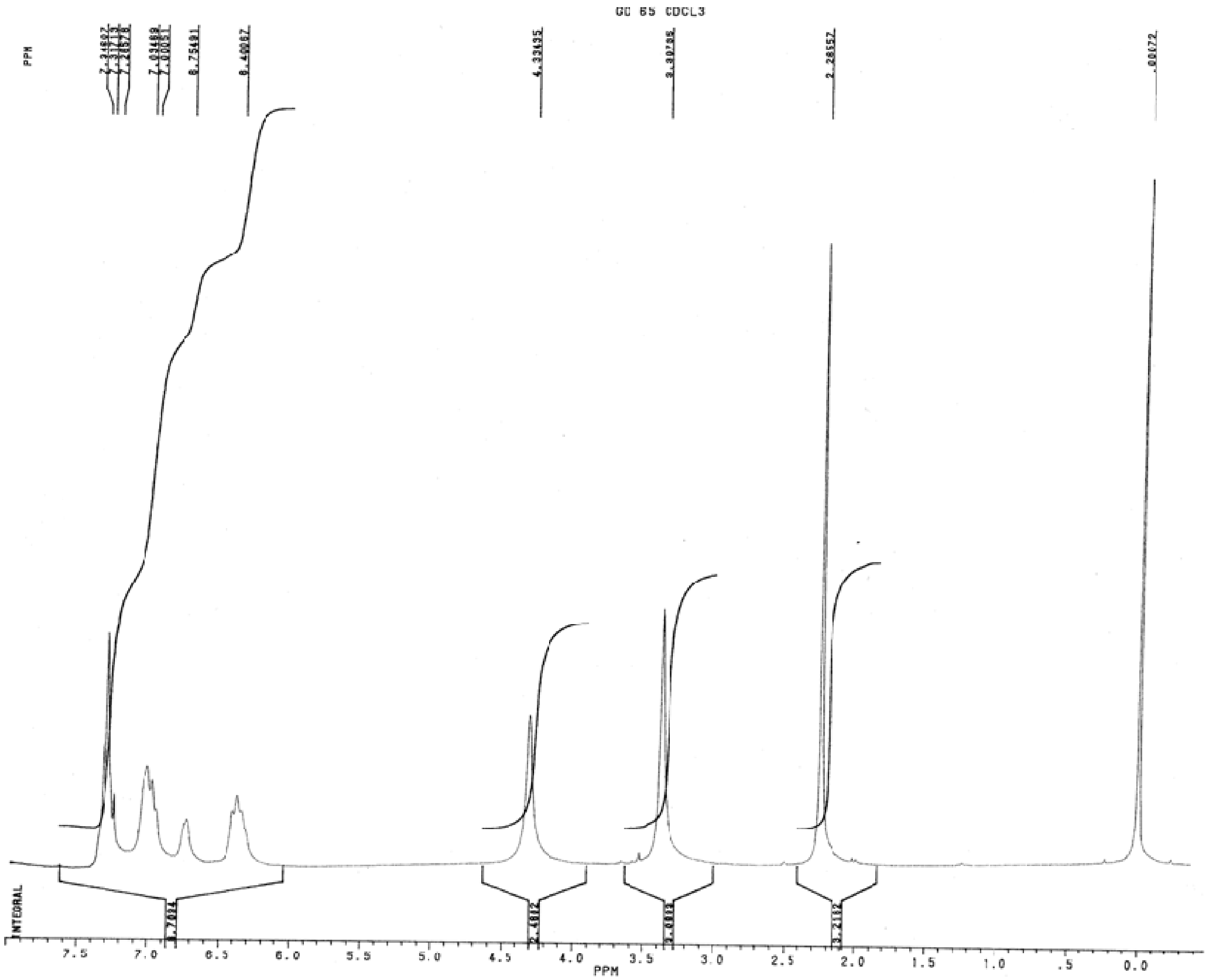

Figure S6. ^{13}C-NMR spectrum of compounds **28**.

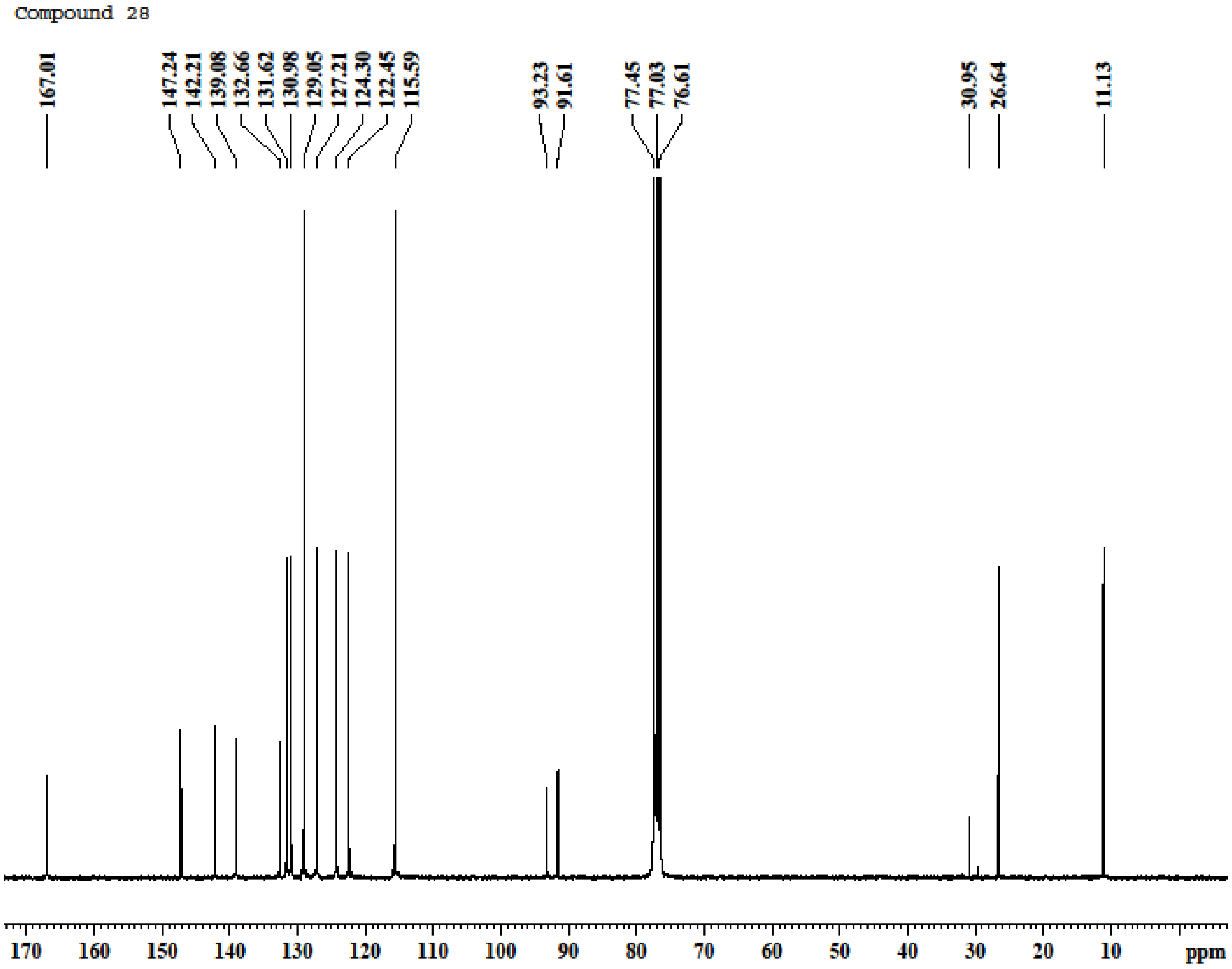

Figure S6. APT spectrum of compounds **28**.

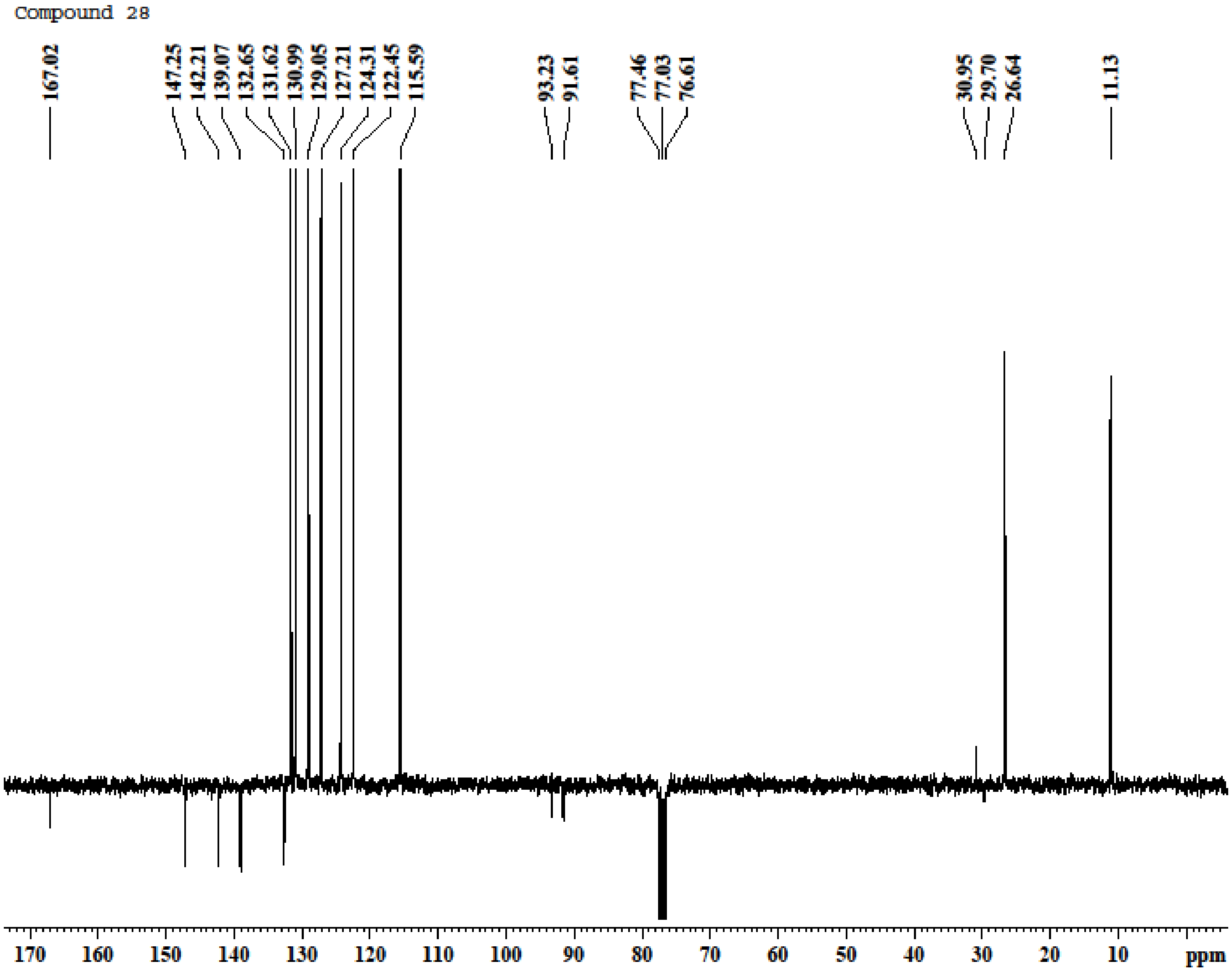

Figure S7. ^{1}H-NMR spectrum of compounds **29**.

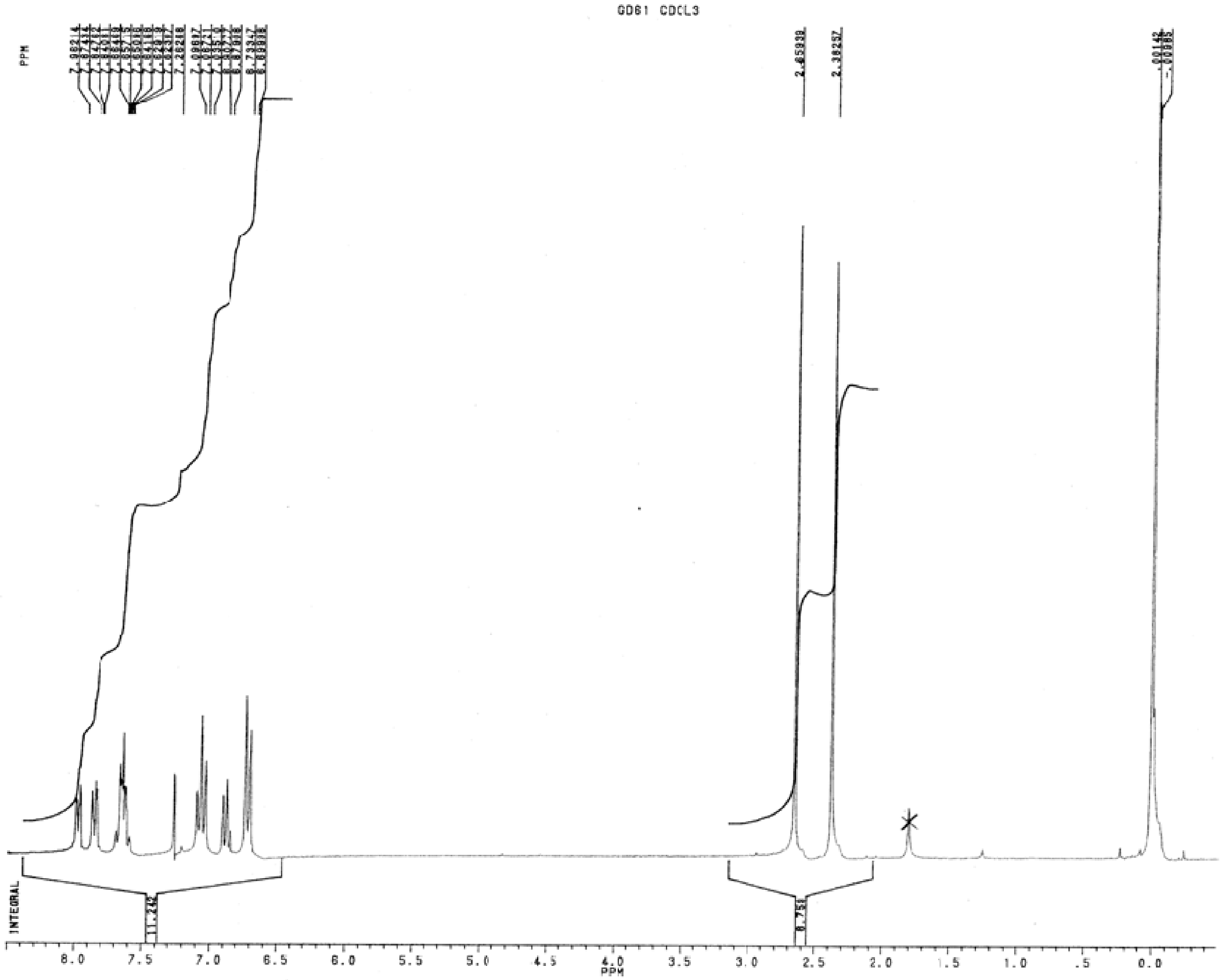

Figure S8. ^{1}H-NMR spectrum of compounds **39**.

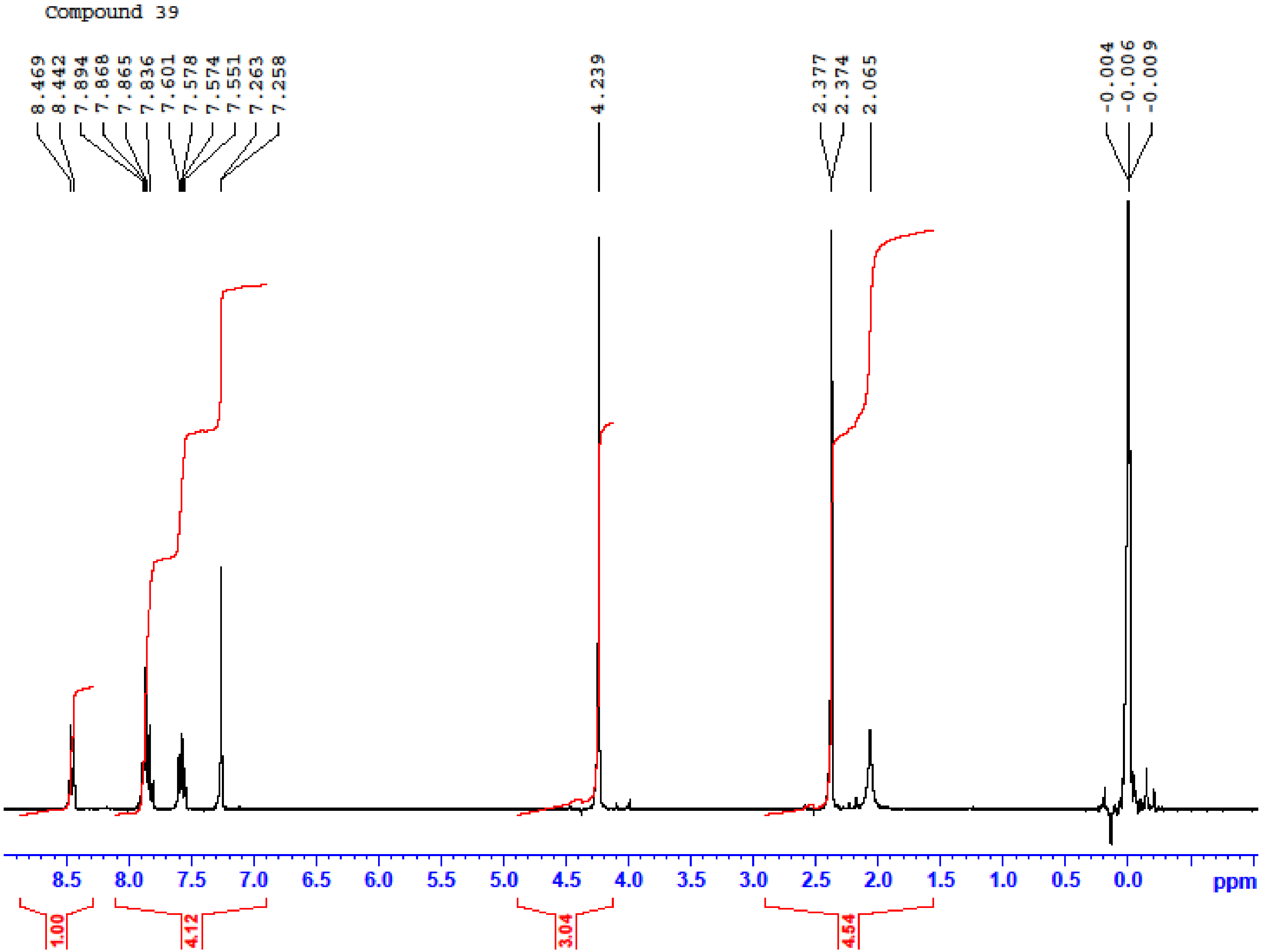

Figure S9. ^{13}C-NMR spectrum of compounds **39**.

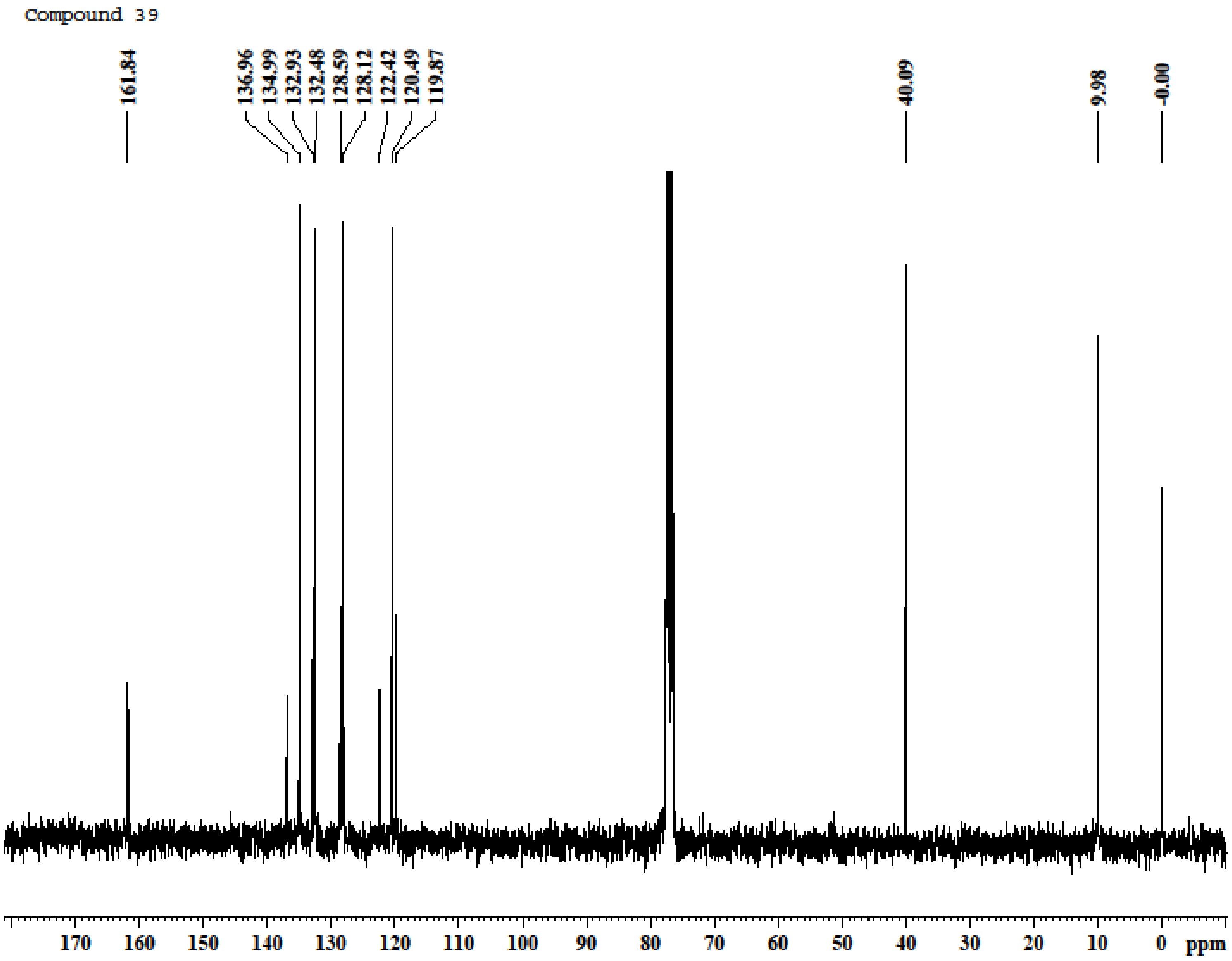

Figure S10. APT spectrum of compounds **39**.

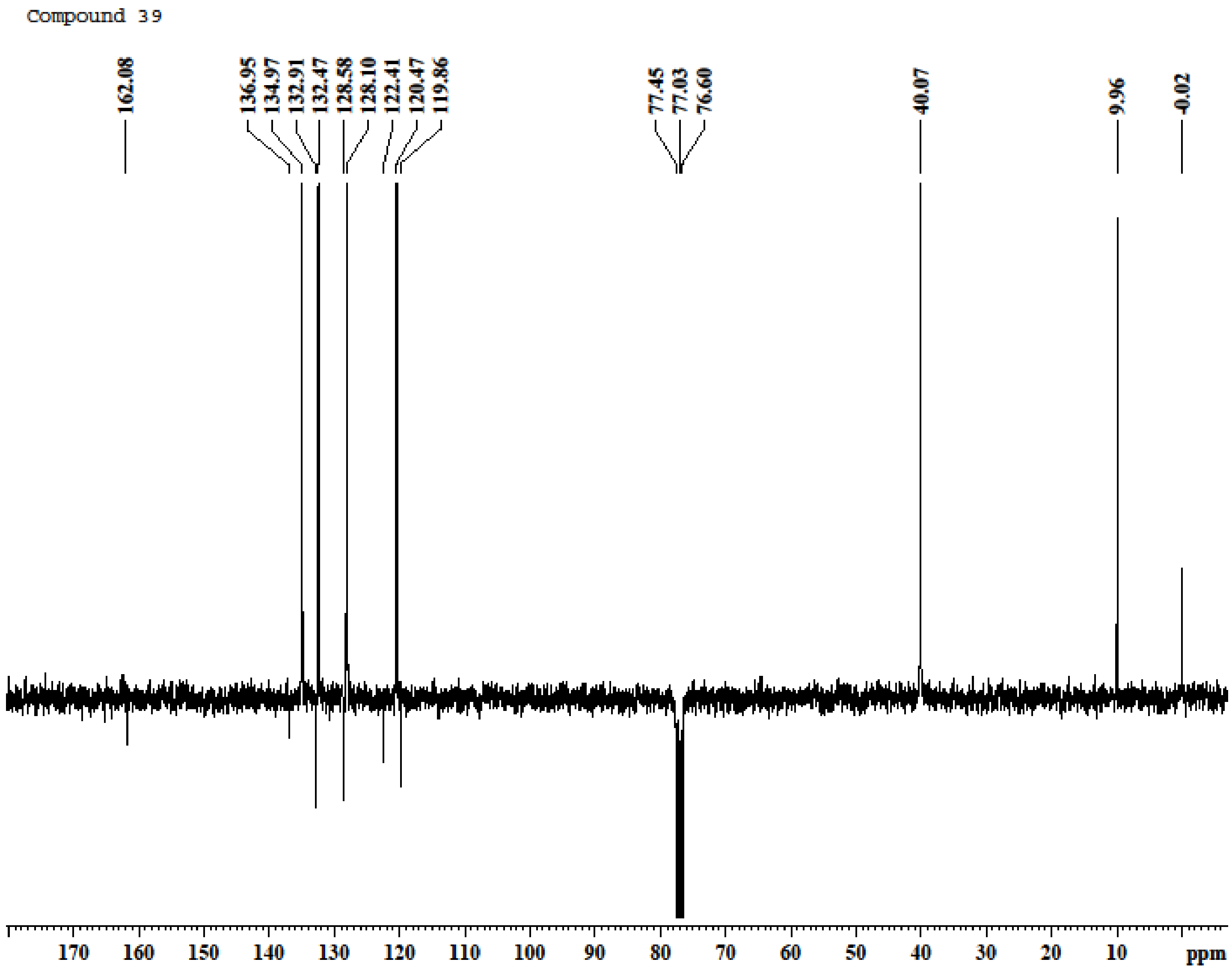

Supplement: Supplementary file 1 [file molecules-18-13096-s001.pdf]
